# Early detection of cerebral ischemia due to pericardium traction using cerebral oximetry in pediatric minimally invasive cardiac surgery: a case report

**DOI:** 10.1186/s40981-019-0273-7

**Published:** 2019-08-17

**Authors:** Fumiaki Hayashi, Rei Nishimoto, Kazuyoshi Shimizu, Tomoyuki Kanazawa, Tatsuo Iwasaki, Hiroshi Morimatsu

**Affiliations:** Department of Anesthesiology and Resuscitology, Okayama University Hospital, 2-5-1 Shikata-cho, Kita-ku, Okayama-shi, Okayama, 700-8558 Japan

**Keywords:** Cerebral ischemia, Near-infrared spectroscopy, Pediatric, Minimally invasive cardiac surgery, Pericardium traction

## Abstract

**Background:**

Minimally invasive cardiac surgery (MICS) for simple congenital heart defects has become popular, and monitoring of regional cerebral oxygen saturation (rSO_2_) is crucial for preventing cerebral ischemia during pediatric MICS. We describe a pediatric case with a sudden decrease in rSO_2_ during MICS.

**Case presentation:**

An 8-month-old male underwent minimally invasive ventricular septal defect closure. He developed a sudden decrease in rSO_2_ and right radial artery blood pressure (RRBP) without changes in other parameters following pericardium traction. The rSO_2_ and RRBP immediately recovered after removal of pericardium fixation. Obstruction of the right innominate artery secondary to the pericardium traction would have been responsible for it.

**Conclusions:**

Pericardium traction, one of the common procedures during MICS, triggered rSO_2_ depression alerting us to the risk of cerebral ischemia. We should be aware that pericardium traction during MICS can lead to cerebral ischemia, which is preventable by cautious observation of the patient.

## Background

Over the last several decades, mortality following congenital heart surgery has been ameliorated because of innovative methodology and performance improvements in equipment used in cardiac surgery [1, 2]. Thus, for better quality of life after congenital heart surgery, it is important to prevent brain dysfunction caused by cerebral ischemia following iatrogenic vessel obstruction during cardiac surgery with cardiopulmonary bypass (CPB). Recently, minimally invasive cardiac surgery (MICS), which is characterized by small skin incision and minimal sternotomy or minithoracotomy, has become popular for use with simple congenital heart surgery and has contributed to good outcomes [3–5]. However, a smaller incision used in MICS makes it more difficult to recognize vessel obstruction leading to cerebral ischemia. Therefore, adequate intraoperative monitoring of brain oxygenation is essential for preventing perioperative cerebral ischemia [6, 7]. Presently, monitoring of regional cerebral oxygen saturation (rSO_2_) has assumed an important role in maintaining proper oxygenation of the brain during pediatric cardiac surgery [6, 7]. Here, we report a pediatric case in which a sudden decline in rSO_2_ alerted us to the possibility of cerebral ischemia due to arterial malperfusion following pericardium traction.

## Case presentation

The patient was an 8-month-old male (height 65.7 cm; weight 7 kg) born through normal vaginal delivery weighing 2768 g. A heart murmur was audible, and the patient was diagnosed as having a perimembranous ventricular septal defect (VSD) with pulmonary hypertension due to a left-to-right shunt 4 days after birth. Transthoracic echocardiography (TTE) showed that the defect passed from the perimembranous septum to the infundibular septum. The defect was 7.4–8.9 mm in diameter and was accompanied by mild tricuspid regurgitation (TR) and mitral regurgitation. The peak TR pressure gradient was 54 mmHg, and the left ventricle ejection fraction was 73%. The patient had no other congenital heart malformations. Although the patient was administered diuretics after diagnosis, he had poor weight gain after 5 months of age, secondary to heart failure. Therefore, the patient was scheduled for VSD closure with lower partial sternotomy.

Preoperative data were within normal ranges, with the exception of the concentration of hemoglobin (Hb) in blood ([Hb]) that was 10.2 g/dL. Upon arrival to the operating room, general anesthesia was induced with 5% sevoflurane in a mixture of 50% nitrous oxide and 50% oxygen. Following placement of an intravenous catheter, endotracheal intubation was facilitated with 70 μg fentanyl and 10 mg rocuronium. Anesthesia was maintained with 1–5% sevoflurane in the air, with concomitant fentanyl and rocuronium as required. During CPB, fentanyl, midazolam, and rocuronium were intravenously administered. Monitoring included electrocardiography and measurement of invasive right radial artery blood pressure (RRBP) and right femoral arterial blood pressure (RFBP), oxygen saturation of peripheral artery (SpO_2_) in the patient’s left finger and toe, end-tidal carbon dioxide (E_T_CO_2_), and central venous pressure (CVP) via a central venous catheter inserted into the right internal jugular vein as well as nasopharyngeal and bladder temperatures. In addition to transesophageal echocardiography (TEE; iE33 xMATRIX S8-3 t Micro TEE transducer; Philips Japan, Ltd.), rSO_2_ was measured by using an INVOS 5100C (Medtronic Japan Co., Ltd.) adult sensor placed on the patient’s right forehead. The insertion of the TEE probe did not affect the patient’s vital signs. The patient’s vital signs at the start of the operation were as follows: RRBP 82/44 mmHg, RFBP 91/52 mmHg, SpO_2_ 100% at fraction of inspired oxygen (FiO_2_) 0.21, E_T_CO_2_ 36 mmHg, CVP 10 mmHg, rSO_2_ 59%, nasopharyngeal temperature (T_naso_) 36.4 °C, and [Hb] 10.2 g/dL.

**Fig. 1:**
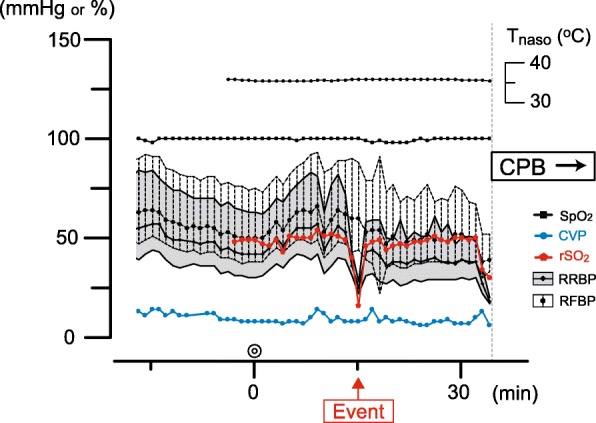
Changes in parameters monitored during general anesthesia around the event of rSO_2_ depression. The graph represents changes of each parameters monitored every minute during general anesthesia from the beginning of the operation to the period just before initiation of cardiopulmonary bypass (CPB). The parameters are as follows: right radial blood pressure (RRBP), right femoral blood pressure (RFBP) and central venous pressure (CVP), oxygen saturation of peripheral artery (SpO_2_), values of regional cerebral oxygen saturation (rSO_2_) measured with INVOS 5100C, and nasopharyngeal temperature (T_naso_). Some of these points beyond measurement limits are considered noise and were thus excluded

Following lower partial sternotomy, the pericardium was towed cephalad and fixed to expose the aorta. Suddenly, RRBP decreased from 82/44 to 26/22 mmHg, followed by a subsequent decrease in rSO_2_ from 59 to 16% (Fig. 1). Simultaneously, the other vital signs remained steady as follows: RFBP 88/44 mmHg, SpO_2_ 100% on the left hand (FiO_2_ 0.21), E_T_CO_2_ 36 mmHg, CVP 10 mmHg, and T_naso_ 36.3 °C. We confirmed that [Hb] was 10.2 g/dL without any signs of sudden hemorrhage in the surgical area. On the basis of these parameters, the decreases of RRBP and rSO_2_ might have been caused by an obstruction of the right innominate artery, secondary to the pericardium and aortic root traction. We promptly discussed this issue with the surgeons and reached a consensus to remove the pericardium fixation. Following removal, RRBP increased to 59/33 mmHg and rSO_2_ to > 50%. Transient reduction in RRBP and rSO_2_ continued for approximately 30 s until the pericardial fixation was removed. Subsequently, the traction of pericardium was modified to maintain rSO_2_ stability. After modification, the operation proceeded uneventfully. Postoperatively, the patient was transferred to the intensive care unit (ICU) under sedation and intubated. The patient gained consciousness and was extubated 3 h later, without any neurological abnormalities. The patient was discharged from the ICU on postoperative day 3.

## Discussion

In this case, pericardium traction, one of the common procedures during MICS, triggered a decrease in rSO_2_ that alerted us to the risk of cerebral ischemia. The change in rSO_2_ was sufficient to cause us to speculate that cerebral ischemia might have occurred in the patient.

**Fig. 2:**
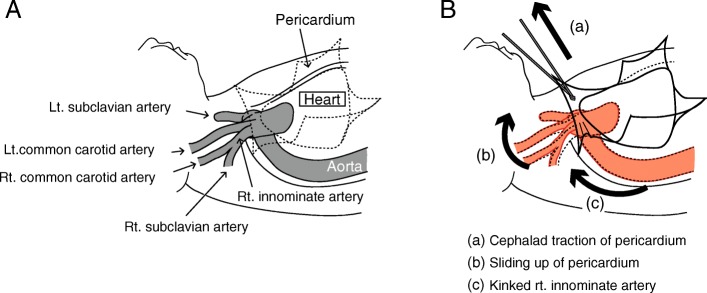
Schematic of our hypothesis. Anatomical images of the aortic root and the pericardium (**A**) and obstruction of the right innominate artery by pericardium traction (**B**). Cephalad traction of the pericardium (a) moves the aortic root to the cephalad ventral direction (b) and obstructs the right innominate artery (c). Rt, right; Lt, left

Recently, a smaller incision during median sternotomy has become an alternative technique during cardiac surgeries for simple congenital heart deformities, such as closure of VSD and atrial septum defects [3, 5]. The smaller incision in pediatric MICS forces the surgeon to pull up the pericardium more strongly to secure a field of view, which can subsequently deform the aorta or the branch (Fig. 2B), which would possibly lead to cerebral ischemia. Additionally, it is extremely difficult to visualize such a physical deformity of arteries by TEE in pediatric MICS. Hence, monitoring of cerebral saturation by near-infrared spectroscopy (NIRS) is becoming an alternative for avoiding such an unforeseen emergency. Some groups have reported that the decrease in rSO_2_ was related to the surgical procedure in cardiac surgery [8, 9], whereas few reports have shown that simple traction of the pericardium can affect rSO_2_ values as we experienced.

Accumulating studies show that the rSO_2_ values measured by NIRS are trustworthy physiological indicators during pediatric cardiovascular surgeries [6, 7]. An interventional algorithm to prevent cerebral desaturation using NIRS has been introduced as a feasible strategy in clinical situations [7, 10, 11]. Following that algorithm, we concluded that arterial malperfusion was plausible because of the following observations: (1) the RRBP and rSO_2_ values dropped simultaneously, (2) SpO_2_ on the left hand remained unchanged, (3) the RFBP was maintained, (4) there was no apparent hemorrhage, (5) the CVP did not change, and (6) the patient’s heart rate and body temperature remained stable. Our decision to insert two artery catheters at the right radial artery and right femoral artery and monitor SpO_2_ at the left hand and foot may have been excessive. However, we aimed to monitor the blood flow at all the extremities during pediatric MICS in order to be alert to any signs indicating obstruction of blood flow. It ultimately gave us more clues to understand what happened in this emergent situation and for future reference.

There are limitations to the use of NIRS devices for monitoring of cerebral saturation. In the present case, we chose the INVOS sensor that reflects a mixture of Hb saturation in venous, capillary, and arterial blood [7]. Alternatively, NIRO®, another device for monitoring cerebral saturation, can give us detailed information from each source [12]. Another issue to be considered is that we used an adult sensor to measure the rSO_2_ value. Although standard values of rSO_2_ vary depending on the type of sensor used [13, 14], they are enough to be trustworthy [6, 7]. In this case, we did not check bilateral brain saturation, but monitoring of RRBP and SpO_2_ on the left hand provided us enough information. A carotid artery ultrasound and/or Transcranial Doppler ultrasound may be preferable for detecting cerebral malperfusions.

In conclusion, we experienced a pediatric case of MICS in which pericardium traction, one of the common procedures in MICS, caused a decrease in rSO_2_, but we were able to prevent cerebral ischemia during the surgery with useful monitoring including rSO_2_, arterial blood pressure, and SpO_2_ at extremities. To achieve better quality of life after congenital heart surgery, we should be aware that pericardium traction during MICS can lead to cerebral ischemia that is preventable by cautious observation of the patient.

## Data Availability

Not applicable.

## Abbreviations

CVP: Central venous pressure
ECG: Electrocardiography
E_T_CO_2_: End-tidal carbon dioxide
FiO_2_: Fraction of inspired oxygen
Hb: Hemoglobin
ICU: Intensive care unit
MICS: Minimally invasive cardiac surgery
NIRS: Near-infrared spectroscopy
RFBP: Right femoral arterial blood pressure
RRBP: Right radial arterial blood pressure
rSO_2_: Regional cerebral oxygen saturation
SpO_2_: Oxygen saturation of peripheral artery
T_naso_: Nasopharyngeal temperature
TR: Tricuspid regurgitation
VSD: Ventricular septal defect
